# Synthesis and Chemical Characterisation of Some New Diheteroaryl Thienothiophene Derivatives

**DOI:** 10.3390/molecules16097706

**Published:** 2011-09-08

**Authors:** Yahia Nasser Mabkhot, Abdullah Mohammad Al-Majid, Abdullah S. Alamary

**Affiliations:** Department of Chemistry, Faculty of Science, King Saud University, P.O. Box 2455, Riyadh 11451, Saudi Arabia

**Keywords:** bis-heterocycles, DMF-DMA, bis-pyrimidine, bis-pyrazole, bis-triazolo pyrimidine

## Abstract

Treatment of 1-(5-acetyl-3,4-dimethythieno[2,3-b]thiophene-2yl)ethanone (**1**) with dimethylformamide dimethyl acetal afforded enaminone derivative **2**, which reacted with amino derivatives to give the corresponding bis-pyrimidine, bis-pyrazole, bis-triazolo-pyrimidine and bis-benzoimidazopyrimidine derivatives.

## 1. Introduction

In the last 30 years, annulated heterocyclic systems have attracted considerable attention both from a theoretical standpoint and in view of their various practical applications [1,2,3,4,5,6,7,8,9,10,11,12,13,14]. Enaminones are valuable intermediates in synthetic organic chemistry [15,16,17,18], and Mabkhot and others [19,20,21,22,23,24,25,26] have reported a variety of syntheses of heteroaromatics developed using functionally substituted enaminones as readily obtainable building blocks possessing multiple electrophilic and nucleophilic moieties. This study was undertaken in continuation of our interest in the chemical and biological properties of thienothiophene derivatives [27,28,29] and our work aimed at the synthesis of a variety of heterocyclic systems for biological and pharmacological evaluation, we have found that 1-(5-acetyl-3,4-dimethylthieno[2,3-b]-thiophene-2-yl) ethanone (**1**) is a versatile, readily accessible building block for the synthesis of several new bis-heterocyclic compounds. 

## 2. Results and Discussion

**Scheme 1:**
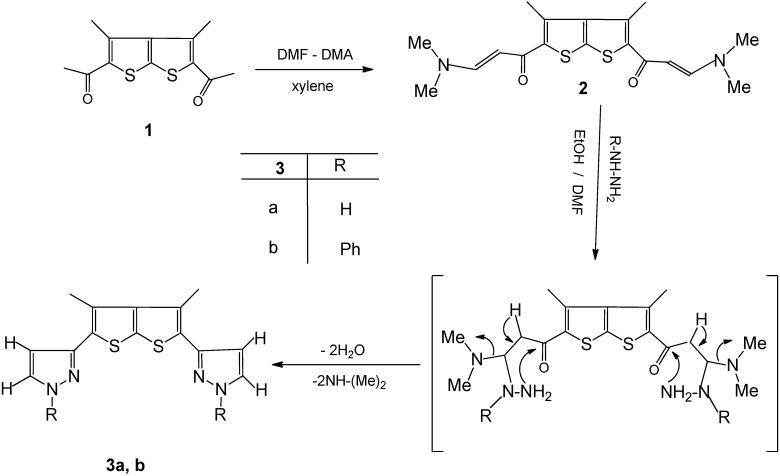
Synthesis of enaminone **2** and pyrazole thienothiophene derivatives **3a,b**.

Treatment of 1-(5-acetyl-3,4-dimethythieno[2,3-b]thiophene-2-yl)ethanone (**1**) with dimethylformamide dimethylacetal (DMF-DMA) in refluxing ethanol afforded 1,1***'***-(3,4-dimethylthieno[2,3-b]thiophene-2,5-diyl)-bis(3(dimethylamino)prop-2-en-1-one (**2**) in high yield (Scheme 1). The ^13^C-NMR spectrum of compound **2**, revealed ten carbon types. The ^1^H-NMR spectrum displayed a singlet at δ 2.22 due to methyl protons, a singlet at δ 2.82 due to the *N,N*-dimethyl protons and at δ 5.36, 5.40 (d, 2H, CH, *J* = 16), 7.62, 7.66 (d, 2H, CH, *J* = 1) due to olefinic protons. The mass spectrum revealed a molecular ion peak at *m/z* 363, corresponding to C_18_H_22_N_2_O_2_S_2_. When compound **2** was treated with hydrazine hydrate or phenylhydrazine in refluxing ethanol/DMF the novel products **3a,b** were obtained, respectively, which then undergo intramolecular cyclization and subsequent aromatization via the loss of dimethylamine and water molecules (Scheme 1). The structures of the latter products were deduced from their elemental analyses and spectral data. The **^1^**H-NMR spectrum of compound **3a**, for example, revealed signals at δ 5.47 (d, 2H, CH, *J* = 5.5), 7.86 (d, 2H, CH, *J* = 5.5) and 13.20 characteristic of pyrazole CH protons and a NH proton, respectively.

**Scheme 2:**
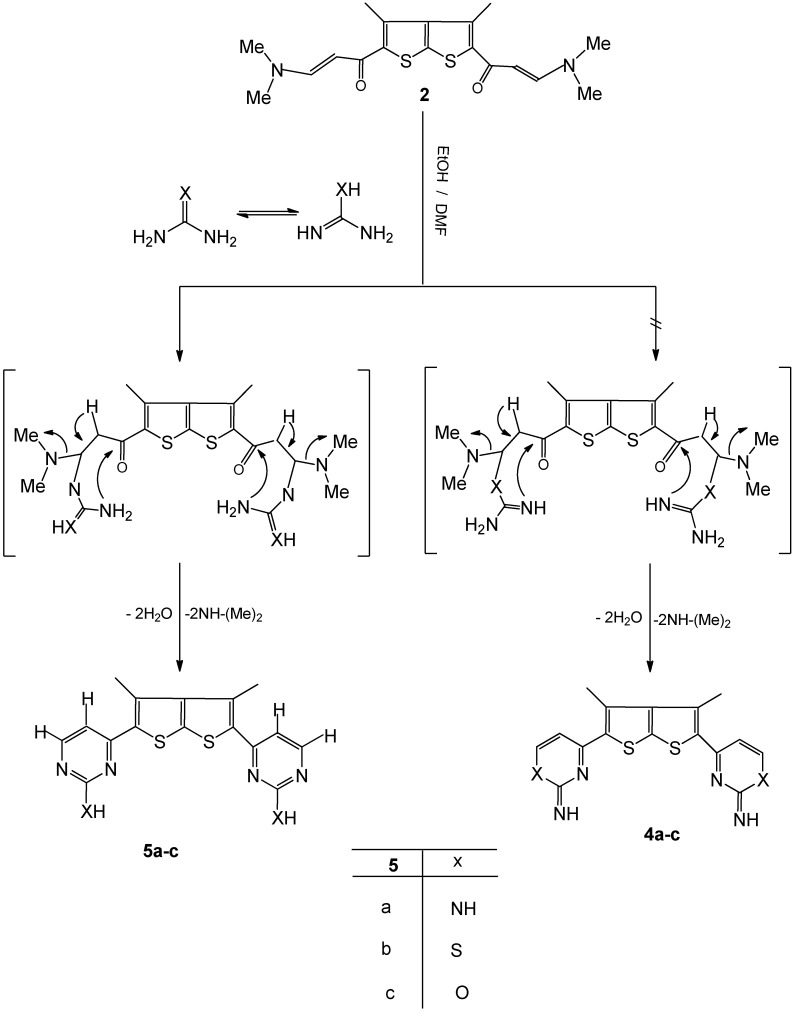
Synthesis of pyrimidine thienothiophene derivatives **5a-c**.

When compound **2** was treated with guanidine, thiourea and or urea in refluxing EtOH/DMF, the the expected derivatives **4a-c** were not obtained, and rather the novel bis-thienothiophene derivatives **5a-c** were formed, which then undergo intramolecular cyclization and subsequent aromatization via the loss of dimethylamine and water molecules under the reaction conditions to give **5a-c**, as depicted in Scheme 2.

The structures of the latter products were deduced from their elemental analyses and spectral data. The IR spectrum of compound **5a**, for example, showed, the absence of carbonyl bands and revealed the appearance of bands in the 3,417 and 3,100 cm^−1^ region due to NH_2_ groups. The structure of product **5a** was confirmed by the ^1^H-NMR spectrum, which displayed a new pair of doublet signals at δ 7. 83, 8.37 with *J* = 12 Hz corresponding to pyrimidine CH protons, as reported for such *E*-coupled protons [30,31,32]. The ^1^H-NMR spectrum also revealed one singlet corresponding to a methyl group at δ 2.21, in additionto the NH_2_ protons at δ 4.83 in Scheme 2. The formation of compound **5a** would involve an initial addition of the amino group in guanidine to the activated double bond in enaminone derivative **2**, followed by deamination to an intermediate which then undergoes cyclization and aromatization via loss of water affording the final isolable product (Scheme 2).

**Scheme 3:**
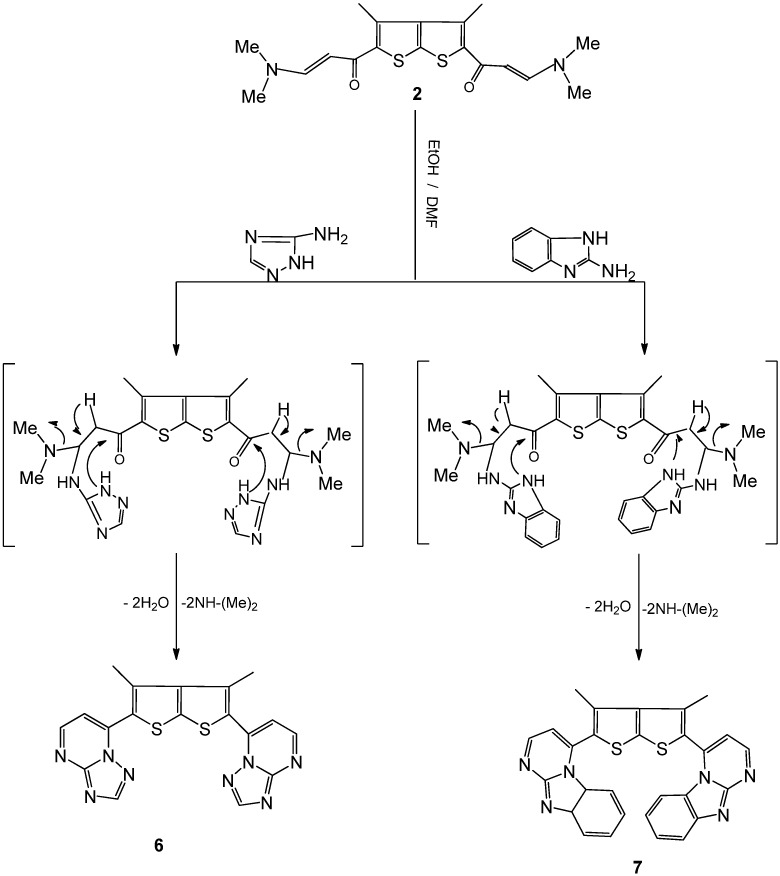
Synthesis of triazolo and benzoimidazolo pyrimidine thienothiophenederivatives **6** and **7**.

The compound 7,7'-(3,4-dimethylthieno[2,3-b]thiophene-2,5-diyl) bis-[1,2,4]triazolo[1,5-a] pyrimidine (**6**) was formed initially via Michael type addition followed by elimination of dimethylamine and water molecules when treatment of compound **2** with 5-amino-1,2,4-triazole in refluxing ethanol/DMF afforded in (Scheme 3).

In the ^1^H-NMR spectra of compound **6** the CH proton appeared as a pair of doublets at 7.66/7.69 ppm (d, 2H, CH, *J* = 12 Hz), and 9.09/9.12 ppm (d, 2H, CH, *J* = 12 Hz) due to vicinal coupling with the two magnetically non-equivalent protons of the methylene group at position 5 and 6 of the pyrimidine ring. Also, the ^1^H-NMR spectrum showed one singlet corresponding to a methyl group at δ 2.24, in addition to the CH proton of triazole at δ 8.62. The mass spectrum revealed a molecular ion peak at *m/z* 404, corresponding to C_18_H_12_N_8_S_2_. In a similar manner, when **2** was treated with 2-aminobenzimidazole, the corresponding compound **7** was obtained in high yield.

## 3. Experimental

### 3.1. General

All melting points were measured on a Gallenkamp melting point apparatus. The infrared spectra were recorded in potassium bromide disks on a Pye Unicam SP 3300 or Shimadzu FT IR 8101 PC infrared spectrophotometers. The NMR spectra were recorded on a Varian Mercury VX-400 NMR spectrometer. ^1^H spectra were run at 400 MHz and ^13^C spectra were run at 75.46 MHz in dimethyl sulphoxide (DMSO-*d_6_*). Chemical shifts were related to that of the solvent. Mass spectra were recorded on a Shimadzu GCMS-QP 1000 EX mass spectrometer at 70 e.V. Elemental analyses were carried out at the Microanalytical Center of King Saud University, Riyadh, Saudi Arabia.

*1,1'-(3,4-Dimethylthieno[2,3-b]thiophene-2,5-diyl)bis(3-(dimethylamino)prop-2-en-1-one* (**2**): A mixture of compound **1** (10 mmol, 0.252 mg) and DMF-DMA (20 mmol, 5 mL) in 99.9% EtOH (20 mL) was refluxed for 8 h, then left to cool to room temperature. The reddish-brown precipitate was filtered off, washed with petroleum ether, and dried. Recrystallization from DMF/EtOH afforded the enaminone derivative **2** in 97% yield, mp. 270–272 °C; IR 1620 (C=O) 1546 (C=C) cm^−1^; ^1^H-NMR δ 2.22 (s, 6H, 2CH_3_), 2.82 (12H, 2CH_3_), 5.36, 5.40 (d, 2H, CH, *J* = 16), 7.62, 7.66 (d, 2H, CH, *J* = 16); ^13^C-NMR δ 15.6, 44.3, 90.4, 136.0, 138.6, 141.5, 147.7, 186.9, 155.3; MS *m/z* (%): 363 (M+1, 37), 362 (M, 100), 347 (84), 318 (10), 284 (6), 98 (100). Anal. Calcd for C_18_H_22_N_2_O_2_S_2_ (362.51); C, 59.64; H, 6.12; N, 7.73; S, 17.69. Found: C, 59.58; H, 5.82; N, 7.44; S, 17.39.

### 3.2. General Procedure for the Reaction of Compound ***2*** with Hydrazine Derivatives

Treatment of compounds **2** (1 mmol) with hydrazine hydrate or phenyl hydrazine (0.1 mL) in dry ethanol (20 mL) under reflux for **7** h afforded the corresponding derivatives **3a** and **3b**, respectively. The solid products were collected by filteration, washed with ethanol, dried and recrystallized from DMF/EtOH.

*3,3'-(3,4-Dimethylthieno[2,3-b]thiophene-2,5-diyl)bis(1H-pyrazole)* (**3a**): White crystals; yield 92%; mp > 320 °C; IR 3186 (NH), 1549 (C=N) cm^−1^; ^1^H-NMR δ 2.10 (s, 6H, 2CH_3_), 5.47, 5.50 (d, 2H, CH, *J* = 5.5), 7.83, 7.86 (d, 2H, CH, *J* = 5.5), 13.20 (2H, pyrazole N-H); ^13^C-NMR δ 14.3, 103.2, 127.2, 130.2, 133.5, 145.7, 148.3, 161.9; MS *m/z* (%): 301 (M+1, 24), 300 (M, 100), 284 (19), 233 (68). Anal. Calcd for C_14_H_12_N_4_S_2_ (300.40); C, 55.97; H, 4.03; N, 18.65; S, 21.35. Found: C, 55.67; H, 3.73; N, 18.38; S, 21.05.

*3,3'-(3,4-Dimethylthieno[2,3-b]thiophene-2,5-diyl)bis(1-phenyl-1H-pyrazole)* (**3b**): Red crystals; yield (87%); mp > 320 °C; IR 1568 (C=N) cm^−1^; ^1^H-NMR δ 2.18 (s, 6H, 2CH_3_), 6.28, 7.16, 7.42 (5H, ArH’s), 6.77, 6.80 (d, 2H, CH, *J* = 12), 8,20, 8.23 (d, 2H, CH, *J* = 12); ^13^C-NMR δ 15.5, 105.5, 120.3, 121.8, 123.6, 125.4, 128.1, 131.5, 135.1, 146.8, 149.3, 162.1; MS *m/z* (%): 453 (M+1, 91), 452 (M, 100), 437 (39), 375 (25). Anal. Calcd for C_26_H_20_N_4_S_2_ (452.11); C, 69.00; H, 4.45; N, 12.38; S, 14.17. Found: C, 68.70 H, 4.18; N, 12.08; S, 13.88.

### 3.3. General Procedure for the Reaction of Compound ***2*** with Guanidine, Thiourea and Urea

Treatment of compound **2** (1 mmol) with guanidine, thiourea or urea (2 mmol) after making sure they dissolve in DMF (2 mL) in dry ethanol (20 mL, 99.9%), under reflux for 6-8 h. afforded the corresponding derivatives **5a-c** respectively. After the solid products were collected by filtration, washed with ethanol, dried and recrystallized from DMF/EtOH.

*4,4'-(3,4-Dimethylthieno[2,3-b]thiophen-2,5-diyl)bis(pyrimidine-2-amine)* (**5a**): Brown crystals; yield 71%; mp. 304–306 °C; IR 3417, 3326 (NH_2_), 1556 (C=N) cm^−1^; ^1^H-NMR δ 2.21 (s, 6H, CH_3_), 4.83 (s, 4H, NH_2_), 7.83 (d, 2H, CH, *J* = 5.5 Hz), 8.37 (d, 2H, CH, *J* = 5.5 Hz); ^13^C-NMR δ 14.92, 102.6, 126.9, 134.1, 135.9, 144.5, 148.2, 158.5, 162.1; MS *m/z* (%): 355 (M+1, 7), 354 (M, 18), 339 (21), 322 (16). Anal. Calcd for C_16_H_14_N_6_S_2_ (354.07); C, 54.22; H, 3.98; N, 23.71; S, 18.09. Found: C, 53.92 H, 3.68; N, 23.68; S, 17.82.

*4,4'-(3,4-Dimethylthieno[2,3-b]thiophen-2,5-diyl)bis(pyrimidine-2-thiol)* (**5b**): Dark yellow crystals; Yield (88%); mp. > 320 °C; IR 3282 (SH), 1622 (C=N) cm^−1^; ^1^H-NMR δ 2.28 (s, 6H, CH_3_), 7.12, (d, 2H, CH, *J* = 5.5 Hz), 8.35 (d, 2H, CH, *J* = 5.5 Hz), 11.82 (2H, SH); ^13^C-NMR δ 16.3, 107.7, 125.6, 136.3, 137.1, 144.8, 149.1, 159.7, 161.1; MS *m/z* (%): 389 (M+1, 67), 388 (M, 78), 354 (6), 277 (31). Anal. Calcd for C_16_H_12_N_4_S_4_ (388.55); C, 49.46; H, 3.11; N, 14.42; S, 33.01. Found: C, 49.93; H, 2.82; N, 14.32; S, 32.92.

*4,4'-(3,4-Dimethylthieno[2,3-b]thiophen-2,5-diyl)bis(pyrimidine-2-ol)* (**5c**): Dark brown crystals; yield 79%; mp > 320 °C; IR 3480 (OH), 1587 (C=N) cm^−1^; ^1^H-NMR δ 2.26 (s, 6H, CH_3_), 8.18(d, 2H, CH, *J* = 5.5 Hz), 8.42 (d, 2H, CH, *J* = 5.5 Hz), 12.62 (2H, OH); ^13^C-NMR δ 15.9, 112.5, 126.3, 134.8, 136.1, 145.6, 147.4, 160.4, 162.5; MS *m/z* (%): 357 (M+1, 58), 356 (M, 18), 355 (2.5), 261 (11). Anal. Calcd for C_16_H_12_N_4_O_2_S_2_ (356.42); C, 53.92; H, 3.39; N, 15.72; S, 17.99. Found: C, 54.00; H, 3.55; N, 15.64; S, 17.78.

### 3.4. General Procedure for the Synthesis of Compounds ***6*** and ***7***

Compound **2** (0.362 g, 1 mmol) in dry DMF (2 mL) was added to 4-amino-1,2,4-triazole (2 mmol, 0.168 gm) or 2-aminobenzimidazole (2 mmol, 0.266 mg), respectively, in dry 99.9% ethanol (20 mL) under reflux for 6–7 h. Then the solid product were collected by filtration, washed with ethanol, dried and recrystallized from (DMF/EtOH) to give **6** or **7**.

*7,7'-(3,4-Dimethylthieno[2,3-b]thiophene-2,5-diyl)bis-[1,2,4]triazolo[1,5-a]pyrimidine* (**6**): Light yellow crystals; yield 88%; mp > 320 °C; IR 1548, (C=N) cm^−1^; ^1^H-NMR δ 2.24 (s, 6H, 2CH_3_),7.66, 7.69 (d, 2H, CH, *J* = 12), 9.09, 9.12 (d, 2H, CH, *J* = 12), 8.62 (2H, =CH, triazole); ^13^C-NMR δ 16.3, 117.2, 128.8, 134.3, 142.0, 145.8, 148.2, 158.7,159.3, 162.1; MS *m/z* (%): 406 (M+2, 41), 405 (M+1, 56), 404 (100), 389 (14), 285 (31). Anal. Calcd for C_18_H_12_N_8_S_2_ (404.06); C, 53.45; H, 2.99; N, 27.70; S, 15.86. Found: C, 53.44; H, 2.81; N, 27.76; S, 15.67.

*2,2'-(3,4-Dimethylthieno[2,3-b]thiophene-2,5-diyl)bis(benzo[4,5]imidazo[1,2-a]pyrimidine)* (**7**): Dark yellow crystals; yield 82%; mp > 320 °C; IR 1529 (C=N) cm^−1^; ^1^H-NMR δ 2.18 (s, 6H, 2CH_3_), 7.56, 7.59 (d, 2H, CH, *J* = 12), 8.37, 8.40 (d, 2H, CH, *J* = 12), 8.12, 8.86 (4H, CH, pyrimidine); ^13^C-NMR δ 15.81, 100.0, 112.1, 115.1, 122.5, 127.1, 131.3, 135.9, 139.1, 142.0, 148.0, 148.4, 156.0, 162.78; MS *m/z* (%): 503 (M+1, 67), 502 (M, 100), 487 (9), 334 (12). Anal. Calcd for C_28_H_18_N_6_S_2_ (502.61); C, 66.91; H, 3.61; N, 16.72; S, 12.76. Found: C, 66.86; H, 3.57; N, 16.86; S, 12.61

## 4. Conclusions

In summary, the reactivity of 1-(5-acetyl-3,4-dimethythieno[2,3-b]thiophene-2-yl)ethanone (**1**) as a versatile and readily accessible building block for the synthesis of new bis-heterocycles incorporating thieno[2,3-b]thiophene was investigated.
